# Low-Cost 3D Systems: Suitable Tools for Plant Phenotyping

**DOI:** 10.3390/s140203001

**Published:** 2014-02-14

**Authors:** Stefan Paulus, Jan Behmann, Anne-Katrin Mahlein, Lutz Plümer, Heiner Kuhlmann

**Affiliations:** 1 Institute of Geodesy and Geoinformation (IGG)—Geodesy, University of Bonn, Nussallee 17, Bonn 53115, Germany; E-Mail: heiner.kuhlmann@uni-bonn.de; 2 Institute of Geodesy and Geoinformation (IGG)—Geoinformation, University of Bonn, Meckenheimer Allee 172, Bonn 53115, Germany; E-Mails: behmann@igg.uni-bonn.de (J.B.); pluemer@igg.uni-bonn.de (L.P.); 3 Institute for Crop Science and Resource Conservation (INRES)—Phytomedicine, University of Bonn, Nussallee 9, Bonn 53115, Germany; E-Mail: amahlein@uni-bonn.de

**Keywords:** low-cost sensors, 3D imaging, David laser scanning system, Microsoft Kinect, parameterization, close-up scanning

## Abstract

Over the last few years, 3D imaging of plant geometry has become of significant importance for phenotyping and plant breeding. Several sensing techniques, like 3D reconstruction from multiple images and laser scanning, are the methods of choice in different research projects. The use of RGBcameras for 3D reconstruction requires a significant amount of post-processing, whereas in this context, laser scanning needs huge investment costs. The aim of the present study is a comparison between two current 3D imaging low-cost systems and a high precision close-up laser scanner as a reference method. As low-cost systems, the David laser scanning system and the Microsoft Kinect Device were used. The 3D measuring accuracy of both low-cost sensors was estimated based on the deviations of test specimens. Parameters extracted from the volumetric shape of sugar beet taproots, the leaves of sugar beets and the shape of wheat ears were evaluated. These parameters are compared regarding accuracy and correlation to reference measurements. The evaluation scenarios were chosen with respect to recorded plant parameters in current phenotyping projects. In the present study, low-cost 3D imaging devices have been shown to be highly reliable for the demands of plant phenotyping, with the potential to be implemented in automated application procedures, while saving acquisition costs. Our study confirms that a carefully selected low-cost sensor is able to replace an expensive laser scanner in many plant phenotyping scenarios.

## 1. Introduction

The importance of automated plant phenotyping has been addressed in many publications in recent years [1–3]. To improve the accuracy and efficiency of phenotyping processes, different types of sensor techniques, such as 3D laser scanning techniques, RGB-cameras, hyperspectral and thermal cameras or chlorophyll fluorescence imaging, have been introduced [2,4,5]. In this context, the characterization of plant features, like the morphology, physiology and performance of a genotype under specific environmental conditions, is expected to increase the efficiency of plant breeding [6].

One important parameter of a plant's phenotype is the shape in three dimensions—the plant architecture—which reflects the adaption of a plant to environmental conditions, like sun intensity, wind or water availability [7,8]. Furthermore, the plant shape contains useful information about the developmental stage during the vegetation period or about yield forming parameters, such as the the current volume of plant fruits or relevant plant organs [9]. However, reliable statements about plant responses to environmental conditions are only possible if a significant number of plants is included in a study [10–12]; thus, sensor techniques with the potential for an implementation in high throughput routines are required.

Commercial systems for accessing the plant shape are based on regular RGBcameras [10,12]. With these systems, it is possible to assess parameters with high speed and very robustly, like the projected plant area or the convex hull spanned by the leaves in 2D. However occlusions and the projection in a 2D image plane remove important parts of the information and fail to exploit the full potential of shape analysis [13]. The full 3D shape overcomes these limitations, but it is expensive in measurement and analysis, so far [9,14,15]. Current developments in sensors and algorithms are mostly driven by the game industry and recently by 3D printing. With this driving force in research and practical use of 3D low-cost systems, the limits of applications (regarding accuracy and resolution) have been redefined [16].

In the agricultural context, laser scanning devices were commonly used for, e.g., kinematic in-field scanning of pear trees [17], 3D modeling of the canopy of tomato plants using different points of view [18] or for the estimation of biomass in different crops [13,19]. In these applications, a very coarse measuring is sufficient. To get a more detailed view on the properties of plants, highly resolved and highly accurate laser scanners are requisite for the observation of the smallest structures [20] and deformation effects, such as wilting [21]. Thus, there is a compelling demand for low-cost 3D imaging techniques for plant phenotyping platforms. However, as stated by [22], there is still a trade-off between the efficiency of image analysis and the costs for a sensor system with adequate plant trait extraction accuracies.

Taking all these factors into account, two low-cost 3D imaging sensors were compared with respect to typical phenotyping scenarios in the present study. The exploited phenotyping tasks cover plant parameter assessment, such as: (a) length, area and volume of sugar beet taproots; (b) sugar beet leaves; and (c) wheat ears.

As low-cost sensors, the Microsoft Kinect [23] and the David laser scanning system [24] were analyzed and compared. The Kinect was included in this study, due to its huge popularity, especially in the robotic and the computer vision communities [25–27]. Furthermore, the first studies dealing with the 3D shape of plants are already available [28,29]. The David system was primarily chosen, due to its build-up by real low-cost components (laser pointer, camera and one calibration corner) with a convincing accuracy [30] and, secondly, due to its wide user domain within universities and the industry. As a reference sensor, a laser scanner (Perceptron V5 scanner coupled to a Romer Infinite 2.0 articulated measuring arm) with an accuracy of 45 μm and a resolution of 17 μm was used [9]. This sensor system is superior to the tested low-cost systems in terms of accuracy, resolution and completeness of the resulting mesh.

During the present study, we aim to emphasize the strengths and drawbacks of both sensor systems within a plant phenotyping task. The prospects and limitations of low-cost sensors and their significance for plant parametrization will be discussed and evaluated.

## 2. Material and Methods

### 2.1. Measuring Systems

Two low-cost laser scanning systems with different measurement principles were compared to a well-established and highly accurate sensor system. A detailed description of the tested devices and the reference system, including prices, is given in the following paragraphs and listed in Table 1.

#### 2.1.1. Microsoft Kinect

**Figure 1.**
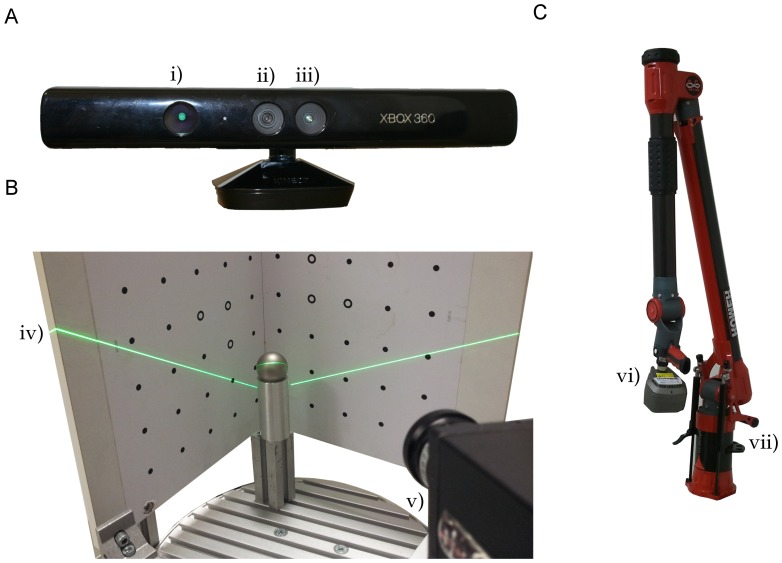
Different 3D sensor systems used in this comparative study: the Kinect (**A**) using an IRemitter (i), a RGBcamera (ii) and the IR camera (iii); the David scanning system (**B**) with the calibration field and the line laser plane (iv) imaged by a David RGB camera (v), as well as the reference system; (**C**) a Perceptron laser triangulation sensor (vi) coupled to a Romer articulated measuring arm (vii).

The Kinect sensor, originally designed for natural interaction in computer gaming environments [25], is a low-cost range sensor. The sensor unit consists of an infrared projector, an infrared camera and a RGB camera (Figure 1A). It captures a depth image with 640 × 480 pixels by a structured light approach at a measurement rate of 30 frames per second (Table 1). For the projection, an infrared laser beam is split into a defined pattern, which is recorded by the infrared camera. A disparity image is calculated [31] by comparison to a previous calibrated reference pattern. Factory-supplied calibration parameters enable the extraction of a point-wise distance values as the distance between the sensor and the object by triangulation.

The accuracy of this depth image has been investigated by several groups and similar to many other optical sensors, it is mainly influenced by the distance between the sensor and the object [31]. The Kinect is originally capable of measuring objects within the distance of 0.8 m to 4 m using the Microsoft software development kit (SDK) [23], but this measurement range may differ using alternative third-party SDKs [32]. With increasing distance, the accuracy decreases from a standard deviation (SD) of a few millimeters to about 4 cm [25], and the point-to-point distance decreases from 0.9 mm to 7 mm, due to the horizontal aperture angle of around 57° [23,31].

For static objects with moderate dynamics, the high frame rate produces a huge amount of redundant information, which can be used to eliminate outliers and to improve the accuracy [31]. The transformation between two frames can be estimated by aligning the respective point clouds. This enables the generation of dense point clouds of real 3D models in contrast to the 2.5D models extracted from the depth images.

Various approaches to extract a consistent 3D model out of the depth video stream were developed [16,32,33]. We used the ReconstructMe console version 0.6.0–405 [34], a software whose underlying tracking algorithm is unpublished. It follows the principles of the well-known KinectFusion algorithm [33] to generate a meshed 3D point cloud from the depth video stream. Single depth images or raw point clouds are not available, as the algorithm generates a meshed surface model directly. In the implemented measurement setup, the object is placed on a rotary disc, and the Kinect is positioned within a distance of around 0.6 m. During the measurement, the 3D model is reconstructed on the graphics processing unit (GPU) by aggregating the different views. The density of the point cloud is decoupled from the sensor resolution and now specified by the software. Further investigations and all measurements were carried out at the meshed point cloud.

#### 2.1.2. David Laser Scanning System

The David laser scanning system (DAVID Vision Systems GmbH, Koblenz, Germany) is a low-cost scanning system [24] consisting of a line laser pointer, a printed calibration field (Type CP-SET01, size DinA3) and a camera (Table 1; Figure 1B). The David USB-CCDmonochrome camera with XGAresolution (1, 024 × 768 pixels), 30 frames per second and a 6 mm prime lens was used. The laser pointer is focusable, battery-driven, has a laser wavelength of 660 nm and is fanned out to a laser line by a cylinder lens. All measurements were controlled by the David software. The exposure time was set to $\frac{1}{100}\sec$, and no smoothness filtering was applied to the sensor data. The processing pipeline can be described by: (1) calibration of the camera using the calibration corner; (2) illuminating the object by the laser line in a ∼45° angle to the viewing direction of the camera; (3) calculating the intersection between the laser plane and the ray from the optical center of the camera to the pixel; and finally, (4) derivation of the 3D coordinates for every pixel illuminated by the laser line. A prerequisite is that the laser line is always visible on both sides of the calibration panel.

Shifting the laser plane vertically enables an illumination of the complete object. The measuring scene could be reconstructed using 30 frames per second. The David system is able to capture the shape of solid and unmoved objects from a single viewpoint. For the full 3D shape, the surface parts need to be referenced by the David software registration routines [24]. As *a priori* knowledge, the angle of the rotation was provided, specifying a rotation only around the vertical axis. The output of the software is a meshed 3D point cloud that combines single scans from different viewpoints.

#### 2.1.3. Perceptron v5 and Romer Infinite 2.0

A comparison study of different 3D low-cost laser scanners needs a reliable validation measurement. For this purpose, a commercial 3D laser triangulation system was used with a line laser scanner (Perceptron Scan Works V5, Perceptron Inc., Plymouth, MI, USA), coupled to an articulated measuring arm (Romer Infinite 2.0 (1.4 m), Hexagon Metrology Services Ltd., London UK; Figure 1C; Table 1). The measuring combination has been proven regarding its applicability for scanning the geometry of tree roots [35], grapevine and wheat [9]. The system has an accuracy of 45 μm within a 2D scanning field with a depth of 110 mm and a mean width of 105 mm. This scanning field is manually moved over the surface of the object. The single scan lines were combined automatically to a complete and almost occlusion-free 3D model of an object. The point cloud was meshed using Geomagic Studio 12 (Raindrop Geomagic Inc, Morrisville, NC, USA).

### 2.2. Data Processing

After scanning the object, all 3D point clouds were processed by the same workflow. All scans were performed under controlled laboratory conditions to avoid the influences of the environment, e.g., wind. Points belonging to the background were cut off using Geomagic Studio 12. The plant parameters, volume, area and diameter, were derived by Geomagic 12. Projections, ear length and ear volume measurements were estimated by procedures using MATLAB 2013a (The MathWorks Inc. Natick, MA, USA).

#### 2.2.1. Error Measurements

The root mean square error (RMSE) [8,13] and the mean absolute percentage error (MAPE) [35,36] were used for error estimation and calculated by the following formulas:

(1) $$\text{RMSE}=\sqrt{\text{mean}{\left(t-a\right)}^{2}}$$

(2) $$\text{MAPE}=\text{mean}\left(\left|\frac{t-a}{t}*100\right|\right)$$

where *t* (target) denotes the reference measurements, *a* (actual) the measured low-cost values and ‖ denotes the absolute value operator.

#### 2.2.2. Accuracy Estimation Using Reference Shapes

The accuracy of the sensors is typically defined by the difference to a test specimen surface. As a test specimen, a steel sphere with a defined diameter of 39.66 mm was used. It provides a diffuse reflecting surface. The planar specimen was made of marble. This provides a high stability and, by coating the surface with scan spray (www.david-vision-systems.de), optimal preconditions for 3D scanning.

**Figure 2.**
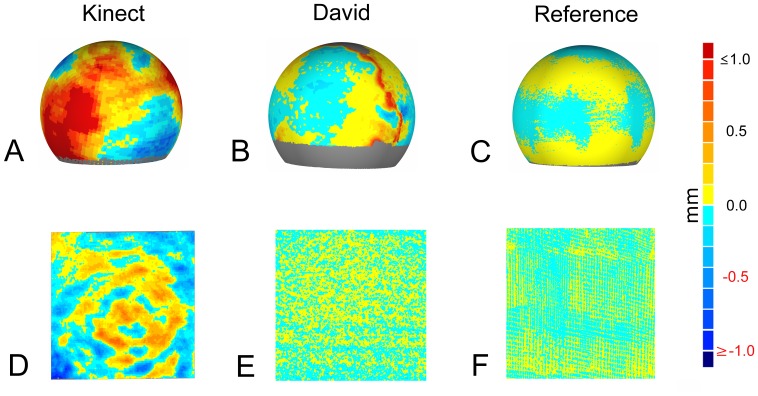
Comparisons of scans of a sphere (**A, B, C**) and a plane (**D, E, F**) with known geometric properties, scanned by the Kinect (**A, D**), the David (**B, E**) and the reference laser scanner (**C, F**). Colors indicate positive (yellow to red) and negative (light blue to dark blue) differences from the original computer-aided design (CAD) model. The grey color indicates parts that are missing due to occlusions.

The target-actual comparison aims at the evaluation of the reproducibility of target geometries by the low-cost sensors. This evaluation had been examined at a spherical and a plane target. For the spherical object, a computer-aided design model (CAD) was available. This was aligned to the scanned point cloud of the Kinect, the David and the reference measurement using iterative closest point (ICP) registration [37]. Geomagic provided a deviation map of the surface, showing differences between the surface points of the scan and the CAD model (Figure 2). This approach is quite common in the literature [25,38]. The target-actual comparison for a plane surface was performed using a planar marble object. A plane was fitted through the measured points, and the differences between measured points and the plane were calculated. The maximum limit for the deviation was set to 1 mm to enable a good visualization.

#### 2.2.3. Plant Parametrization

##### Sugar beet root volume

The volume of the taproots of sugar beet plants was assessed consecutively with the three different measuring systems after harvesting. Ten sugar beet taproots (cultivar Pauletta, KWS, Einbeck, Germany), grown at the field station in Klein-Altendorf (Germany, vegetation period 2012), were measured. Height was defined as the maximal distance between two points when projecting all the points to the vertical axis and width as the maximal distance between two points when projecting the points on the ground plan. Taproot volume and surface were extracted from the 3D triangulation mesh. The parameter compactness was calculated by the quotient of surface and volume.

##### Sugar beet leaves area

One main aspect of 3D plant parameter extraction is the derivation of 3D data of above ground plant organs, such as plant leaves and stems. Based on suitable 3D models, the amount of photosynthetic active area, the leaf orientation, the plant organ formation, as well as plant growth during the vegetation period can be described. Sugar beet plants, variety Pauletta (KWS, Einbeck, Germany), grown under controlled conditions in a greenhouse were measured, and the area of eight leaves was calculated separately from the point clouds. As parameters, the leaf area as a shape in 3D and the projected leaf area using the pre-defined ground plane were assessed.

##### Wheat ears

Yield-forming plant organs, such as ears or fruits, can be directly assessed by 3D sensors. They are important parameters to evaluate the performance of crops or horticultural plants. In the present study, an experiment with wheat plants, variety Taifun (KWS, Lochow, Germany) was undertaken. Wheat plants were cultivated in a greenhouse until ear ripening, and the length and volume of ten ears was extracted from the 3D mesh.

## 3. Results

**Figure 3.**
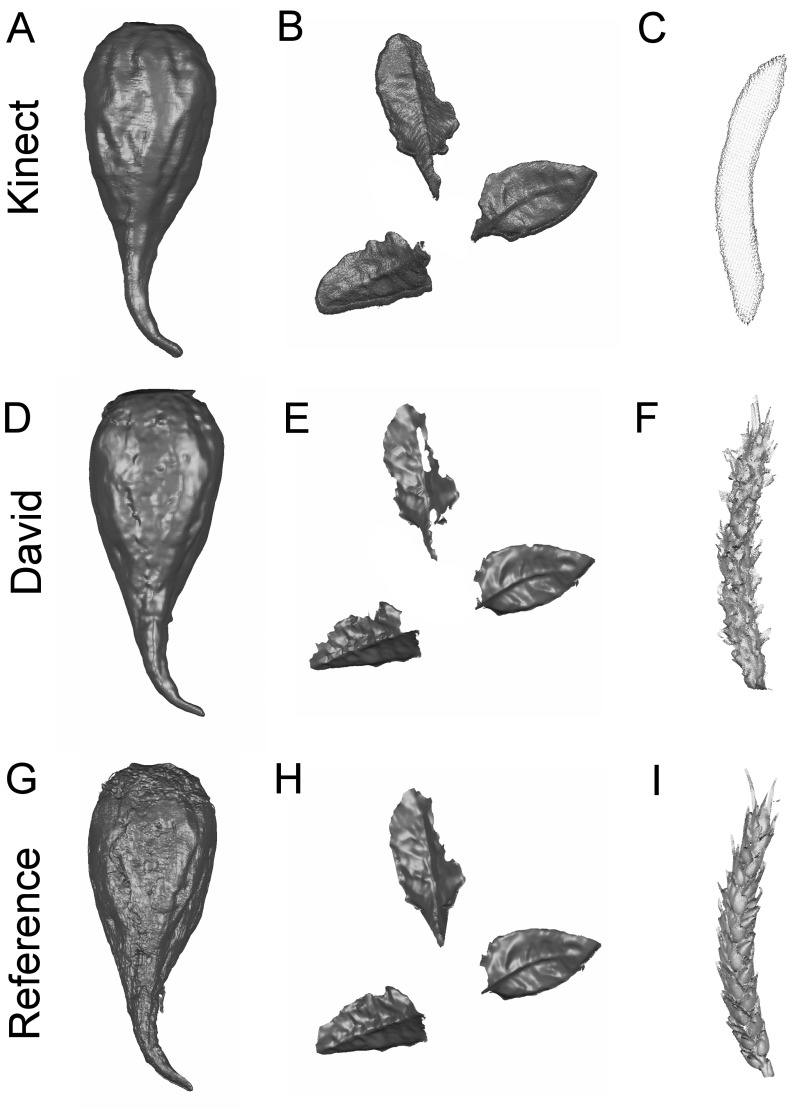
Measuring plant organs with the tested 3D sensors. Rows indicate scans with the same sensor, Kinect (upper row, **A,B,C**), David (middle row, **C,D,E**) and the reference (lower row, **F,G,H).** The meshed scans of a sugar beet taproot are shown (**A,D,G**). The visualization of the meshed point clouds of the sugar beet leaves (**B,E,H**). The measured point clouds of a wheat ear (**C,F,I**).

The potential of the low-cost sensors was tested in different measuring scenarios. The 3D measuring accuracy was estimated based on deviations of the test specimen (Figure 2). Specific plant parameters for plant phenotyping were derived from the volumetric shape of sugar beet taproots, the leaves of sugar beets and the shape of wheat ears (Figure 3) and evaluated.

### 3.1. Definition of Accuracy, Compared to Perceptron

The accuracies of the used sensors were tested with the described specimen. The comparison of the target-actual experiment is visualized in Figure 2. While Figure 2A,B,C shows the target-actual comparison of a sphere scan of Kinect, David and Perceptron, Figure 2D,E,F demonstrates an analogue comparison of the plane measurements. The examined Kinect scans exhibit a standard deviation of 0.70 mm for the sphere specimen and 0.24 mm for the plane (Table 2). Figure 2A shows large areas with high differences (≥1.00 mm) and only a few areas with small deviations. The related scan of a plane (Figure 2D) shows areas of the highest deviation (≤−0.50 mm) with clear peaks concentrically from the middle (≤−1.00 mm). The highest differences were calculated as 1.96 mm and −1.77 mm for the sphere and 0.76 mm and −0.99 mm for the plane. The measurements of the same test objects with the David system showed a standard deviation of 0.18 mm for the sphere and 0.08 mm for the plane (Table 2). The target-actual comparison exhibited differences to a sphere of ≥0.50 mm, especially where different scan views were registered (Figure 2B). The scan of the plane points resulted in the smallest differences (positive and negative) distributed homogeneously over the surface (Figure 2E). The maximal deviations were 0.99 mm and −0.89 mm for the sphere and 0.37 mm and −0.35 mm for the plane, respectively (Table 2). The reference measurement with the Perceptron/Romer scanning combination showed the smallest deviations (positive and negative), resulting in a standard deviation of 0.05 mm for the sphere scan (Figure 2C) and 0.03 mm for the plane scan (Figure 2F). The maximum deviations were 0.17 mm and −0.031 mm for the the sphere scan and 0.11 mm and 0.13 mm for the plane scan.

### 3.2. Comparative Assessment of 3D Parameters of Sugar Beet Taproots

**Figure 4.**
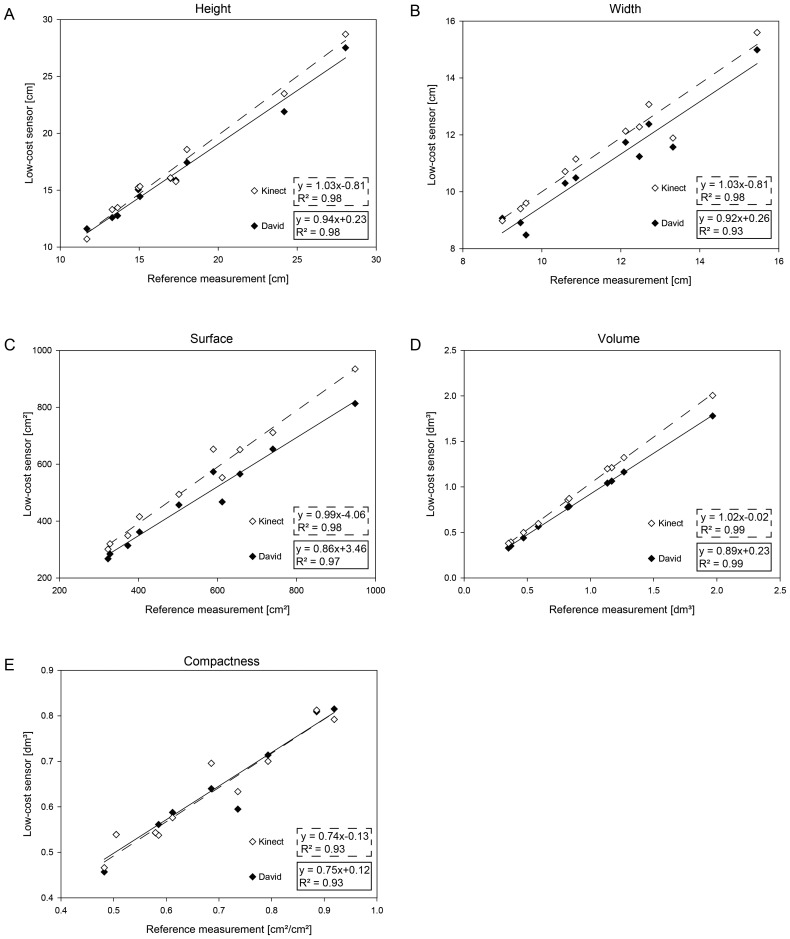
Sugar beet taproot parameter comparison: The extracted parameters for the sugar beet taproot height (**A**), width (**B**), volume (**C**), surface (**D**) and compactness (**E**) as correlations between the low-cost sensors Kinect and David to the reference measurements.

The parameters, width, height, volume, surface and compactness, for sugar beet taproots are presented in Figure 4. A high correlation compared to the reference taproot measurement is shown for all the extracted parameters. The Kinect provided a correlation of *R*^2^ = 0.981 with a RMSE of 0.88 cm for the height, while David had a correlation of *R*^2^ = 0.98 at a RMSE of 1.02 cm (Figure 4A). The measurement of the width provided a correlation of *R*^2^ = 0.98 with a RMSE of 0.66 cm for the Kinect. The David measurements exhibited a correlation of *R*^2^ = 0.93 with a RMSE of 0.83 cm (Figure 4B).

The 3D parameters were highly correlated with a *R*^2^ = 0:99 for both sensors and a RMSE of 0.12 cm and 0.08 cm for the volume measured by the Kinect and by the David low-cost sensor, respectively (Figure 4C). The surface measurements showed a correlation of *R*^2^ = 0:98 with a RMSE of 68.54 cm for the Kinect and of *R*^2^ = 0:97 with a RMSE of 82.10 cm for the David sensor (Figure 4D). The compactness calculated out of the volume and surface parameters were correlated with *R*^2^ = 0:93 with a RMSE of 0.02 cm for the Kinect and *R*^2^ = 0.93 with a RMSE of 0.07 cm for David (Figure 4E). The results exhibit a significant, high correlation throughout the parameters.

### 3.3. Measurement of Sugar Beet Leaves

**Figure 5.**
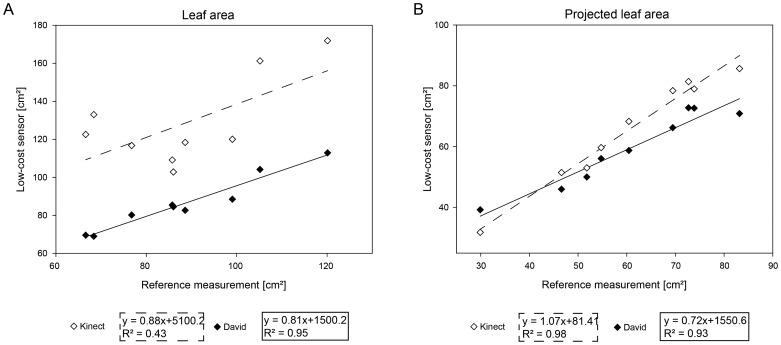
Comparative measurements of sugar beet leaves. (**A**) The correlations of the low-cost systems regarding the leaf area parameter; (**B**) The correlations of the parameter, projected leaf area, of the low-cost systems compared to the reference measurements.

To validate the extraction of leaf parameters, two sugar beet plants were scanned, providing eight leaves of a diverse size. As comparison parameters, the leaf area of every single leaf was extracted, as well as the area of the vertically projected leaves on the ground plane. The results of the comparison are shown in Figure 5. Similar to previous experiments, the accuracy of parameters automatically extracted form the scans of the low-cost sensors were compared to the reference system (Table 3). For the leaf area, the David system showed a high correlation of R^2^ = 0.94 and a RMSE of 4.97 cm^2^. The Kinect systems provided a immense lower correlation result of R^2^ = 0.42 and a RMSE of 43.33 cm^2^ (Figure 5A). When projecting the leaf area to the ground plane, the David system provided a high correlation of R^2^ = 0.93 and a RMSE of 5.35 cm^2^. Similar results were obtained for the Kinect sensor, providing a high correlation of R^2^ = 0.97 and a RMSE of 17.09 cm^2^ (Figure 5B).

### 3.4. Assessment of Wheat Ears as Yield Parameters

**Figure 6.**
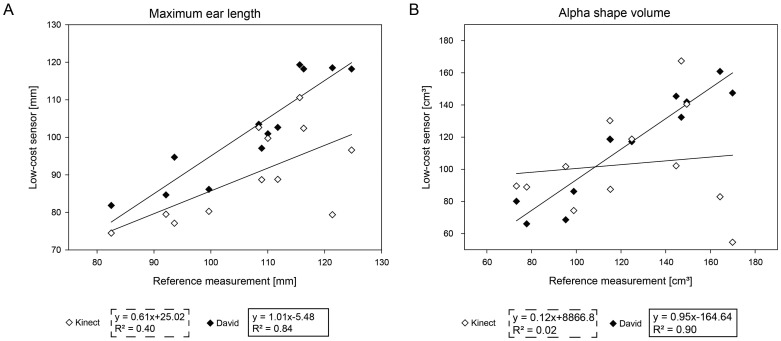
Measuring of ear parameters of a wheat plant. (**A**) The correlation graphic of the low-cost systems compared to the reference measurement regarding the maximum ear length. (**B**) The correlation using the alpha shape volume as the considered parameter.

Wheat ears presented a considerable challenge for the used scanning systems regarding the handling of scattered surfaces and the completeness of scans. Figure 6A presents the correlation of the used low-cost systems to the reference measurement. The Kinect measurements show a correlation of *R*^2^ = 0.83, and the David systems reveals a correlation of *R*^2^ = 0.40. The RMSE error was determined as 17.09 mm for the Kinect system and 6.64 mm for the David system. Figure 6B denotes the correlations for Kinect (*R*^2^ = 0.89) and David (*R*^2^ = 0.02) regarding the alpha shape volume. While the Kinect measurements have a RMSE of 4.32 cm^3^, the David scans come along with a RMSE of 1.09 cm^3^.

## 4. Discussion

Capturing the three-dimensional shape of plants offers access to a variety of geometrical features. The main outcome of the presented study is that the evaluated low-cost sensors do allow an accurate 3D parameterization of various plants, as it is a prerequisite for plant phenotyping (Figure 3 and Tables 4 and 5). A MAPE of less than 10% was reached with at least one of the tested low-cost sensors in all experiments. This limit of tolerance was defined by [39] as the acceptable error rate for morphological plant phenotyping, indicating that the error is low enough to distinguish between two measuring dates during plant development. One of the low-cost sensors fulfilled this demand (Tables 3, 4 and 5) in each of the investigated scenarios. We confirmed the hypothesis that low-cost sensors are well suited for the tracking of phenological plant parameters. The method is probably transferable to other crops and scenarios with similar accurate results. Volumetric objects, like tomatoes, maize and citrus fruits, can be scanned and observed by the Kinect sensor with convincing results. For smaller objects with filigree and scattered surface structures, like wheat ears, grapevine and coffee beans, the David sensor is preferable.

The error distribution in this experiment reveals some limitations and drawbacks (Figure 2): The David system has a drastically decreased accuracy in regions where scans from different points of view had been fused, whereas the Kinect system reaches visually poor results at the small sphere, due to the limited spatial resolution. However, the accuracy of the Kinect sensor was higher than expected by previous publications [25]. Based on the results from the tested objects, it can be concluded that the tested low-cost sensor systems are able to capture the 3D shape of objects depending on their size and complexity. As the next step, the capability of the low-cost systems at different relevant plant phenotyping applications was examined.

The characterization of storage organs is of high importance for sugar beet breeding. The analysis of the sugar beet taproot showed that both sensors are well suited for an adequate 3D parameterization. The parameters height, width, volume, surface and complexity are descriptive for the taproot size, shape and weight. The Kinect system exhibits a high accordance with the reference scans. This is demonstrated by a MAPE of 4.77% of the Kinect and 6.72% of the David system for the volume measurement. The extracted sugar beet taproot characteristics seem to be very robust against smaller inaccuracies and over-smoothing effects (Figure 4). The accurate results of height and width examinations are consistent to those of [28], who found a high correlation of Kinect-based parameters with manual parameter measurements. The David sensor showed slight drawbacks for the estimation of compactness and surface area. This can be attributed to the errors occurring when merging scans from different viewpoints. Important yield and quality parameters of sugar beet varieties can be derived, since the size and shape of the taproot is closely linked to sugar content and quality [40]. Based on these findings, we can conclude that the Kinect sensor is able to achieve better results than the David sensor if the parameter is robust against smoothing and if the object size is sufficient (Table 4).

The leaf area is a relevant parameter for the description of plant growth, development and productivity of monocotyledonous and dicotyledonous plants [4,10]. For leaf measurements, the Kinect reveals severe deficiencies in estimating the 3D leaf area with a MAPE of 47.64%. Strong smoothing effects shift the leaf border up to 10 millimeters beyond the real leaf border and generate a corona around the leaf. The additional surface is twisted away from the sensor, and therefore, the leaf area is drastically overestimated. A projection to a horizontal plane (the so-called projected leaf area) reduces these effects, and the accuracy of the projected leaf area (MAPE = 8.49%) is comparable to the results assessed by the David sensor (MAPE = 6.93%). The projected leaf area, defined as the ground cover, can be used as a proxy for agricultural productivity, since the photosynthetic activity and, thus, the carbon-gain is linked to the leaf area directed to sunlight [41,42]. It allows an estimation of the Leaf Area Index (LAI, [43]) in early stages of development and the identification of the smallest changes in growth rates [44].

In contrast to the taproots, the parameter, leaf area (Table 3), shows a negative effect of smoothing at the leaf margins. A projection on a horizontal plane reduces its impact and provides reliable predictions on plant characteristics. It simplifies the data analysis and supports the comparison to the common data of cameras [44].

The two plant organs, taproot and leaf, are representational examples illustrating that the optimal sensor selection depends on the application.

Measuring wheat ear volumes and geometric parameters allows for the monitoring of the yield development of cereal crops [9,18]. As a function of the number of kernels per ear and the kernel weight [22], cereal yield is a complex agronomic trait. Previous studies demonstrated a high correlation of wheat ear volume to yield, characterizing parameters like the kernel number or the ear weight [9]. However, the measurement of filigree wheat ear structures, consisting of single kernels, awns and husks, reveals the limitations of low-cost scanners regarding the scannable object size. The Kinect removes details (Figure 1) and captures only a small volumetric object (MAPE 24.60%). This affects the maximum length calculation of the single ears and results in a MAPE of 15.54%. In contrast, the David system assesses the volume with a higher, more suitable accuracy (MAPE 7.74%) and the length with a MAPE of 4.89%. The reference scanner determines the 3D shape with high accuracy and the smallest details. Focusing on smaller objects with high curvature, the low resolution of the Kinect sensor causes a strong smoothing effect, and therefore, the shape of the wheat ears cannot be accurately measured (Table 5). A surface that should be measured by the Kinect has to fulfill a specific smoothness constraint, which is not given by the rugged surface of the wheat ears. In these scenarios, the David sensor with a higher resolution is preferable. However, it was demonstrated that both low-cost systems can be used for a reliable plant parameter extraction. Each sensor has different advantages, e.g., scanning field, accuracy, automated fusion and resolution. Before implementation into phenotyping setups, the sensor choice should be adapted to the parameter of interest.

Our study shows the prospects and limitations of each system. 3D imaging systems can help to improve common setups for plant organ parametrization and reduces errors due to occlusions. Existing 3D processing pipelines can be reduced in cost by low-cost sensors [22,39]. However, the highly dynamic development of new sensors may pull the performance far above the current point within months. The upcoming new version of Kinect is promoted to be higher resolved, more accurate and having a wider field of view [45]. The implementation of these sensors for plant phenotyping tasks will give further impulses to the field of low-cost phenotyping. As postulated by [22], the tested low-cost systems will support plant phenotyping processes by providing less expensive and sophisticated data interpretation infrastructures.

We are confident that the presented methods will support plant scientists during high-throughput phenotyping [46] to gain new insights and valuable information for the 3D geometry in the time course (4D) and in the next step to unravel the genetic information encoding relevant plant traits.

## 5. Conclusions

Our study showed the reliability of low-cost data for the parametrization of plant organs in phenotyping setups. Low-cost sensors are able to replace expensive sensors in the presented scenarios. The sensor choice has to be adapted to the scanning scenario regarding the required level of detail, as well as to the measuring field and the smoothness properties of the organ surface. Surfaces of objects, like sugar beet taproots, are preferred to be scanned by the Kinect sensor. For filigree objects, like rugged wheat ears and the surface of leaves, the David sensor is well suited.

Low-cost sensors offer the potential to design phenotyping systems even for small companies and research facilities with limited resources. The shown approach of using low-cost sensors for plant imaging may help to face the limitation defined as the phenotyping bottleneck.

## Figures and Tables

**Table 1.** Overview of the main sensor properties and the prices for the Kinect sensor, the David and the reference method.

	**Lowcost Sensors**		**Reference Method** **Perceptron-Romer**

	**Microsoft Kinect**	**David Laserscanning System**	
cost (12.01.2014)	∼100 €	∼1,000 €	∼100,000 €
resolution	∼0.2% of the object size	depending on measuring setup	17 μm
accuracy	depending on object fragmentation	∼0.1% of the object size	45 μm
used wavelength	827 nm	660 nm	660 nm
measurable volume	distance of 0.5 m to 5 m	depending on the measuring setup	spherical (1.4 m radius)
manual registration	no	yes	no
main application field	human pose detection	simple 3D projects	quality management

**Table 2.** Accuracies of the 3D measurements of the test objects for the different sensors. The maximum positive deviation (MPD), the maximum negative deviation (MND) and the standard deviation (SD) are given for the three sensors and the two test objects.

		**Low-Cost Sensors**		**Reference** **Perceptron-Romer**
		**Kinect**	**David**	
sphere	MPD (mm)	1.96	0.99	0.17
	MND (mm)	1.77	0.89	0.31
	SD (mm)	0.70	0.18	0.05

plane	MPD (mm)	0.76	0.37	0.11
	MND (mm)	0.99	0.35	0.13
	SD (mm)	0.24	0.08	0.03

**Table 3.** Accuracies of the plant parameters, leaf area and projected leaf area, extracted from the 3D geometry of sugar beet leaves measured by Kinect and David. RMSE, root mean square error; MAPE, mean absolute percentage error.

**Parameter**	**Kinect**			**David**		
	***R*^2^**	**RMSE**	**MAPE**	***R*^2^**	**RMSE**	**MAPE**
single leaf area (cm^2^)	0.43	43.33	47.64%	0.95	4.97	4.03%
projected single leaf area (cm^2^)	0.98	5.82	8.49%	0.93	5.35	6.93%

**Table 4.** Accuracies of the plant parameters height, width, volume, surface and compactness extracted from the 3D geometry of sugar beet taproots measured by Kinect and David.

**Parameter**	**Kinect**			**David**		
	***R*^2^**	**RMSE**	**MAPE**	***R*^2^**	**RMSE**	**MAPE**
height (cm)	0.98	0.88	3.59%	0.98	1.02	4.55%
width (cm)	0.98	0.66	2.08%	0.93	0.83	5.61%
volume (dm^3^)	0.99	0.12	4.77%	0.99	0.083	6.72%
surface (cm^2^)	0.98	68.54	4.70%	0.97	82.10	13.16%
compactness $\left(\frac{{\text{cm}}^{2}}{dm^{3}}\right)$	0.93	0.02	7.94%	0.93	0.07	8.21%

**Table 5.** The accuracies of the plant parameters, volume and length, extracted from the 3D geometry of wheat ears measured by Kinect and David.

**Parameter**	**Kinect**			**David**		
	***R*^2^**	**RMSE**	**MAPE**	***R*^2^**	**RMSE**	**MAPE**
alpha shape volume (cm^3^)	0.40	4.32	24.60%	0.84	1.09	7.74%
maximum length (mm)	0.02	17.09	15.54%	0.90	6.64	4.89%

## References

[1] Furbank R., Tester M. (2011). Phenomics–technologies to relieve the phenotyping bottleneck. Trends Plant Sci..

[2] Fiorani F., Schurr U. (2013). Future scenarios for plant phenotyping. Annu. Rev. Plant Biol..

[3] Dhondt S., Wuyts N., Inzé D. (2013). Cell to whole-plant phenotyping: The best is yet to come. Trends Plant Sci..

[4] Rascher U., Blossfeld S., Müller-Linow M., Nagel K., Pieruschka R., Pinto F., Schreiber C., Temperton V., Thorpe M., van Dusschoten D. (2011). Non-invasive approaches for phenotyping of enhanced performance traits in bean. Functional Plant Biol..

[5] Mahlein A., Oerke E., Steiner U., Dehne H. (2012). Recent advances in sensing plant diseases for precision crop protection. Eur. J. Plant Pathol..

[6] Frasson R., Krajewski W. (2010). Three-dimensional digital model of a maize plant. Agric. For. Meteorol..

[7] Fourcaud T., Zhang X., Stokes A., Lambers H., Körner C. (2008). Plant growth modeling and applications: The increasing importance of plant architecture in growth models. Ann. Bot..

[8] Omasa K., Hosoi F., Konishi A. (2007). 3D lidar imaging for detecting and understanding plant responses and canopy structure. J. Exp. Bot..

[9] Paulus S., Dupuis J., Mahlein A., Kuhlmann H. (2013). Surface feature based classification of plant organs from 3D laserscanned point clouds for plant phenotyping. BMC Bioinform..

[10] Berger B., Parent B., Tester M. (2010). High-throughput shoot imaging to study drought responses. J. Exp. Bot..

[11] Granier C., Aguirrezabal L., Chenu K., Cookson S., Dauzat M., Hamard P., Thioux J., Rolland G., Bouchier-Combaud S., Lebaudy A. (2006). PHENOPSIS, an automated platform for reproducible phenotyping of plant responses to soil water deficit in Arabidopsis thaliana permitted the identification of an accession with low sensitivity to soil water deficit. New Phytol..

[12] Hartmann A., Czauderna T., Hoffmann R., Stein N., Schreiber F. (2011). HTPheno: An image analysis pipeline for high-throughput plant phenotyping. BMC Bioinform..

[13] Keightley K., Bawden G. (2010). 3D volumetric modeling of grapevine biomass using Tripod LiDAR. Comput. Electron. Agric..

[14] Vos J., Evers J., Buck-Sorlin G., Andrieu B., Chelle M., de Visser P. (2010). Functional–structural plant modeling: A new versatile tool in crop science. J. Exp. Bot..

[15] El-Omari S., Moselhi O. (2011). Integrating automated data acquisition technologies for progress reporting of construction projects. Autom. Constr..

[16] Rusu R., Cousins S. 3D is Here: Point Cloud Library (PCL).

[17] Palacín J., Pallejà T., Tresanchez M., Sanz R., Llorens J., Ribes-dasi M., Masip J., Arnó J., Escolà A., Rosell J. (2007). Real-time tree-foliage surface estimation using a ground laser scanner. Instrumentation.

[18] Hosoi F., Omasa K. (2009). Estimating vertical plant area density profile and growth parameters of a wheat canopy at different growth stages using three-dimensional portable lidar imaging. ISPRS J. Photogr. Remote Sens..

[19] Busemeyer L., Ruckelshausen A., Möller K., Melchinger A., Alheit K., Maurer H., Hahn V., Weissmann E., Reif J., Würschum T. (2013). Precision phenotyping of biomass accumulation in triticale reveals temporal genetic patterns of regulation. Sci. Rep..

[20] Wagner B., Gaertner H., Ingensand H., Santini S. (2010). Incorporating 2D tree-ring data in 3D laser scans of coarse-root systems. Plant Soil.

[21] Cai X., Sun Y., Zhao Y., Damerow L., Schulze Lammers P., Sun W., Lin J., Zheng L., Tang Y. (2013). Smart detection of leaf wilting by 3D image processing and 2D Fourier transform. Comput. Electron. Agric..

[22] Yang W., Duan L., Chen G., Xiong L., Liu Q. (2013). Plant phenomics and high-throughput phenotyping: Accelerating rice functional genomics using multidisciplinary technologies. Curr. Opin. Plant Biol..

[23] Microsoft (2010). Kinect Specifications. http://msdn.microsoft.com/en-us/library/jj131033.aspx.

[24] Winkelbach S., Molkenstruck S., Wahl F., Franke K., Müller K., Nickolay B., Schäfer R. (2006). Low-cost Laser Range Scanner and Fast Surface Registration Approach. Pattern Recognition.

[25] Khoshelham K., Elberink S. (2012). Accuracy and resolution of Kinect depth data for indoor mapping applications. Sensors.

[26] Cui Y., Schuon S., Thrun S., Stricker D., Theobalt C. (2013). Algorithms for 3D shape scanning with a depth camera. IEEE Trans. Pattern Anal. Mach. Intell..

[27] Henry P., Krainin M., Herbst E., Ren X., Fox D. (2012). RGB-D mapping: Using Kinect-style depth cameras for dense 3D modeling of indoor environments. Int. J. Robot. Res..

[28] Azzari G., Goulden M., Rusu R. (2013). Rapid characterization of vegetation structure with a Microsoft Kinect sensor. Sensors.

[29] Chéné Y., Rousseau D., Lucidarme P., Bertheloot J., Caffier V., Morel P., Belin É., Chapeau-Blondeau F. (2012). On the use of depth camera for 3D phenotyping of entire plants. Comput. Electron. Agric..

[30] DAVID 3D Scanning. http://www.david-3d.com/.

[31] Mankoff K., Russo T. (2013). The Kinect: A low-cost, high-resolution, short-range, 3D camera. Earth Surf. Process. Landf..

[32] Whelan T., Kaess M., Fallon M., Johannsson H., Leonard J., McDonald J. Kintinuous: Spatially Extended KinectFusion.

[33] Newcombe R., Davison A., Izadi S., Kohli P., Hilliges O., Shotton J., Molyneaux D., Hodges S., Kim D., Fitzgibbon A. KinectFusion: Real-Time dense Surface Mapping and Tracking.

[34] (2014). ReconstructMe. http://reconstructme.net/.

[35] Wagner B., Santini S., Ingensand H., Gärtner H. (2011). A tool to model 3D coarse-root development with annual resolution. Plant Soil.

[36] Hosoi F., Nakabayashi K., Omasa K. (2011). 3-D Modeling of tomato canopies using a high-resolution portable scanning lidar for extracting structural information. Sensors.

[37] Besl P., McKay N. (1992). A method for registration of 3-D shapes. IEEE Trans. Pattern Anal. Mach. Intell..

[38] Khoshelham K. Accuracy Analysis of Kinect Depth Data.

[39] Paproki A., Sirault X., Berry S., Furbank R., Fripp J. (2012). A novel mesh processing based technique for 3D plant analysis. BMC Plant Biol..

[40] Tsialtas J., Maslaris N. (2010). Sugar beet root shape and its relation with yield and quality. Sugar Tech.

[41] Anten N. (2005). Optimal photosynthetic characteristics of individual plants in vegetation stands and implications for species coexistence. Ann. Bot..

[42] Evans J., Poorter H. (2001). Photosynthetic acclimation of plants to growth irradiance: The relative importance of specific leaf area and nitrogen partitioning in maximizing carbon gain. Plant Cell Environ..

[43] Andrieu B., Allirand J., Jaggard K. (1997). Ground cover and leaf area index of maize and sugar beet crops. Agronomie.

[44] Munns R., James R., Sirault X., Furbank R., Jones H. (2010). New phenotyping methods for screening wheat and barley for beneficial responses to water deficit. J. Exp. Bot..

[45] Microsoft Kinect 2.0 Announcement in Official Blog. http://blogs.msdn.com/b/kinectforwindows/archive/2013/11.aspx.

[46] Busemeyer L., Mentrup D., Möller K., Wunder E., Alheit K., Hahn V., Maurer H., Reif J., Würschum T., Müller J. (2013). BreedVision–a multi-sensor platform for non-destructive field-based phenotyping in plant breeding. Sensors.

